# Endothelial dysfunction and renal fibrosis in endotoxemia-induced oliguric kidney injury: possible role of LPS-binding protein

**DOI:** 10.1186/s13054-014-0520-2

**Published:** 2014-09-27

**Authors:** Giuseppe Castellano, Alessandra Stasi, Angelica Intini, Margherita Gigante, Anna Maria Di Palma, Chiara Divella, Giuseppe Stefano Netti, Clelia Prattichizzo, Paola Pontrelli, Antonio Crovace, Francesco Staffieri, Enrico Fiaccadori, Nicola Brienza, Giuseppe Grandaliano, Giovanni Pertosa, Loreto Gesualdo

**Affiliations:** Nephrology, Dialysis and Transplantation Unit, Department of Emergency and Organ Transplantation, University of Bari, Piazza Giulio Cesare 11, 70124 Bari, Italy; Nephrology, Dialysis and Transplantation Unit, Department of Medical and Surgical Sciences, University of Foggia, Foggia, Italy; Veterinary Surgery Unit, Department of Emergency and Organ Transplantation, University of Bari, Bari, Italy; Department of Clinical Sciences, University of Parma, Parma, Italy; Anesthesia and Intensive Care Unit, Department of Emergency and Organ Transplantation, University of Bari, Bari, Italy

## Abstract

**Introduction:**

The pathophysiology of endotoxemia-induced acute kidney injury (AKI) is characterized by an intense activation of the host immune system and renal resident cells by lipopolysaccharide (LPS) and derived proinflammatory products. However, the occurrence of renal fibrosis in this setting has been poorly investigated. The aim of the present study was to investigate the possible association between endothelial dysfunction and acute development of tissue fibrosis in a swine model of LPS-induced AKI. Moreover, we studied the possible effects of coupled plasma filtration adsorption (CPFA) in this setting.

**Methods:**

After 9 hours from LPS infusion and 6 hours of CPFA treatment, histologic and biochemical changes were analyzed in pigs. Apoptosis and endothelial dysfunction were assessed on renal biopsies. The levels of LPS-binding protein (LBP) were quantified with enzyme-linked immunosorbent assay (ELISA). Endothelial cells (ECs) were stimulated *in vitro* with LPS and cultured in the presence of swine sera and were analyzed with FACS and real-time RT-PCR.

**Results:**

In a swine model of LPS-induced AKI, we observed that acute tubulointerstitial fibrosis occurred within 9 hours from LPS injection. Acute fibrosis was associated with dysfunctional alpha-smooth muscle actin (α-SMA)^+^ ECs characterized by active proliferation (Ki-67^+^) without apoptosis (caspase-3^-^). LPS led to EC dysfunction *in vitro* with significant vimentin and N-cadherin expression and increased collagen I mRNA synthesis. Therapeutic intervention by citrate-based CPFA significantly prevented acute fibrosis in endotoxemic animals, by preserving the EC phenotype in both peritubular capillaries and renal arteries. We found that the removal of LBP from plasma was crucial to eliminate the effects of LPS on EC dysfunction, by blocking LPS-induced collagen I production.

**Conclusions:**

Our data indicate that EC dysfunction might be pivotal in the acute development of tubulointerstitial fibrosis in LPS-induced AKI. Selective removal of the LPS adaptor protein LBP might represent a future therapeutic option to prevent EC dysfunction and tissue fibrosis in endotoxemia-induced AKI.

## Introduction

Sepsis is a complex disease arising from the host response to an overwhelming infection. Gram-negative bacteria and the components of their walls, in particular the lipid A-containing lipopolysaccharide (LPS), play a major role in the pathogenesis of sepsis [1]. As in Gram-negative sepsis, during endotoxemia, LPS induces uncontrolled cytokines release, activation of coagulation on endothelial cells (ECs) [2] leading to shock, multiple organ damage, and even death [3]. During sepsis and endotoxemia, acute kidney injury (AKI) is a frequent complication [2].

The pathophysiology of sepsis-induced AKI is characterized by intrarenal hemodynamic changes, EC dysfunction, infiltration of inflammatory cells in renal parenchyma, intraglomerular thrombosis, and tubular obstruction with necrotic/apoptotic-derived cellular debris [2]. EC dysfunction [4,5] is a term that includes a number of changes defined by profound alterations in EC functions, including transition from a quiescent to an activated state [6]. The activation of EC [7,8] leads to an increased expression of cell-adhesion molecules such as ICAM-1, and chemokines with subsequent enhancement of EC-leukocytes interaction [9]. Interestingly, recent evidence highlighted the direct implications of EC dysfunction in mediating tissue fibrosis by different mechanisms [9].

EC [10] and tubular epithelial cells [11] are activated by LPS through the Toll-like receptor-4 (TLR-4), myeloid differentiation protein-2 (MD-2), and CD14 complex. LPS activation on EC induces both proinflammatory and cytoprotective effects [12]. Conversely, renal tubular epithelial cells undergo apoptotic and necrotic processes [13] when activated by LPS. Extracorporeal treatments based on plasma adsorption have been proposed as a possible approach to modify the course of sepsis-induced AKI, interfering with the imbalance between pro- and antiinflammatory factors induced by LPS [1,2,14].

In this study, we investigated the possible association between EC dysfunction and acute development of tissue fibrosis in a swine model of LPS-induced AKI, and we tested whether citrate-based coupled plasma filtration adsorption (CPFA) therapy might be beneficial in this model.

## Methods

### Animal model

The animal model of endotoxemia was developed in domestic swine at the Faculty of Veterinary Medicine, Bari University, after approval by the ethical committee of the Italian Ministry of Education, University, and Research (MIUR). Female pigs, with a body weight of 58.4 ± 14.7 kg, 6.8 ± 07 months old, were fasted for 24 hours before the experiment. All animals were premedicated with an intramuscular mixture of Telazol (tiletamine + zolazepam) 4 to 5 mg/kg and atropine 0.04 mg/kg (atropine sulfate 0.1%; ATI, Bologna, Italy). After 20 minutes, a 20-gauge catheter was introduced into the auricular vein of the right ear, and an infusion of Ringer lactate solution (LRS) was started (10 ml/kg/h). General anesthesia was induced with an intravenous infusion of IV fentanyl (5 μg/kg) (Fentanest; Pharmacia & Upjohn, Milano, Italy) followed by propofol (3 to 5 mg/kg to effect) and maintained with a constant-rate infusion of propofol (5 to 8 mg/kg/h) and fentanyl (10 μg/kg/h). Additional boluses of fentanyl and propofol were given as needed. After induction of anesthesia, animals were endotracheally intubated by a cuffed tube and connected to a breathing circuit. After intubation, the pigs received an intermittent positive pressure ventilation (IPPV) with a tidal volume and respiratory rate set to maintain end-tidal CO_2_ partial pressure (P_ET_CO_2_) between 30 and 40 mm Hg. All animals received 40% of oxygen through the breathing circuit (Ohmeda 7850 ventilator; Datex Ohmeda, Helsinki, Finland).

An 18-gauge catheter was inserted into the left carotid artery for arterial blood sampling and systemic blood pressure measurement. A triple-lumen 16-gauge central venous catheter was advanced into the left jugular vein for drugs and LRS administration, and for recording of central venous pressure.

Body temperature was maintained by a thermoregulated blanket control unit. A urinary catheter was placed in all animals for the measurement of urine output and for urinalysis. Electrocardiogram (ECG), pulse oximetry, capnography, airways pressures and tidal volume, blood glucose, and lactate were also monitored.

We randomized the animals into four groups: control (CTR, *n* = 7), CPFA (CPFA-treated healthy pigs, *n* = 7), LPS (endotoxemic pigs, *n* = 7), and LPS CPFA (CPFA-treated endotoxemic pigs, *n* = 7).

Through the previously isolated venous access, in LPS and LPS CPFA groups, 10 ml of a saline solution containing 300 μg/kg of LPS (lipopolisaccharide membrane of *Escherichia coli*) was infused. CTR and CPFA pigs received 10 ml of sterile saline solution.

CPFA was performed with plasma filter (input: 25 μm polypropylene; output: 25 μm polypropylene +5 μm nylon; Bellco, Mirandola, Italy) and adsorption cartridge (styrene resin with macroporous structure, 30 nm; Mediasorb Bellco). Blood flow rate (Qb) was included between 100 ml/min and 250 ml/min, ultrafiltration rate at 35 ml/kg/h, and plasma filtration at 30 ml/min. The local citrate anticoagulation protocol was validated by using Lynda equipment. Predilution citrate was infused (citrate bag: Na +136 m*M*, citrate 10 mm, citric acid 2 m*M*). Calcium was infused in postdilution (Na +139 m*M*, K +1.5 m*M*, Ca^2+^ 2 m*M*, HCO_3_, 35 m*M*, and glucose, 5.55 m*M*) [15]. During the CPFA session, blood samples were collected to control calcium systemic levels at 1.05 to 1.2 m*M* and within the circuit at 0.2 to 0.3 m*M*. The duration of treatment was 6 hours. During treatment, hemodynamic and respiratory parameters were continuously monitored. Animals were killed after 9 hours from LPS/saline infusion or after 6 hours CPFA treatment (T9) with an overdose of IV thiopental, immediately followed by a 10-ml IV bolus of an oversaturated solution of potassium chloride (Sigma-Aldrich, Gillingham, UK).

### Collection of samples

A renal biopsy was performed at the start of experimental procedure (T0). Multiple biopsies were then obtained at different intervals from saline or LPS infusion and at death (T9).

A portion of each biopsy specimen was immediately snap-frozen in Optimal Cutting Temperature (OCT, Tissuetek) medium and stored in liquid nitrogen. Another portion was fixed in buffered formalin (4%) for 12 hours and embedded in paraffin by using standard procedures.

Urine samples were collected from all animals, and urinary output was measured and recorded every hour. Swine sera were collected at T0, at intermediate time points, and at T9 from an arterial blood catheter. Plasma samples were drawn from a CPFA circuit at the inlet (plasma pre-cartridge) and outlet (plasma post-cartridge) of the adsorption cartridge 1 hour after installation (T4), at different intervals and finally after 6 hours of treatment (T9). Sera and plasma samples were stored at −80°C until their use.

### Renal-function measurements

Serum Kidney Injury Molecule-1 (KIM-1) and Cystatin C measurements were performed with commercially available enzyme-linked immunoassays (ELISAs; Uscn Life Science Inc, Wuhan, Hubei, China) according to manufacturer’s instructions.

### Cell culture

Immortalized human umbilical vein EC line (EA.hy926), obtained from American Type Culture Collection (ATCC, Rockville, MD, USA) were cultured in DMEM high-glucose medium supplemented with 10% FBS, 100 U/ml penicillin, 0.1 mg/ml streptomycin, 2 m*M* L-glutamine (Sigma Aldrich) at 37°C in an atmosphere of 5% CO_2_. When cells became confluent, they were stimulated for 24 hours with LPS 2 μg/ml and 4 μg/ml and were incubated for 12 hours in the presence of 1% of different swine sera with/without LPS Binding Protein (LBP), 9 μg/ml.

### Masson trichrome staining

Two-μm-thick sections of swine paraffin-embedded renal biopsies were deparaffinized and rehydrated with alcohol. Then they were washed with distillated water and incubated with Bouin solution overnight at room temperature. The slides were stained in Weigert iron hematoxylin solution for 6 minutes. After that, they were washed and stained with scarlet-acid fuchsin solution for 15 minutes. The sections were differentiated in 1% acetic acid solution for 1 minute, and then they were immersed in phosphomolybdic acid for 4 minutes. They were again differentiated in acetic acid solution and immersed in green-light solution 2% for 5 minutes. After differentiation with acetic acid solution, slides were dehydrated, cleared in xylene, and mounted with Eukitt.

Digital slides were then acquired with the Aperio ScanScope CS2 device (Aperio Technologies, Vista, CA, USA) with 20× magnification. Green-stained area was quantified by using Adobe Photoshop software and expressed as positive pixel/total pixel by two independent observers blinded to the origin of the slides. The final quantification was the mean of the two measures. In no case was interobserver variability higher than 20%.

### Confocal laser scanning microscopy

Swine paraffin-embedded renal sections were stained or double stained for α-SMA (Santa Cruz Biotechnologies, Santa Cruz, CA, USA), CD31 (Abcam, Cambridge, MA, USA), and Ki-67 (Novus Biologicals, CA, USA). All the antibodies cross-react with pig tissue. Tissue sections were deparaffinized through xylene and alcohol and underwent epitope retrieval through three microwave (750 W) cycles of 5 minutes in citrate buffer (pH = 6). Then they were incubated with specific blocking solution, primary antibodies (anti-α-SMA 1:100, anti-CD31 1:30, anti-Ki-67 1:50) and the corresponding secondary antibodies (Alexa Fluor 488 goat anti-mouse; AlexaFluor 555 goat anti-rabbit, and AlexaFluor 488 goat anti-rabbit, Molecular Probes, Eugene, OR, USA). All sections were counterstained with TO-PRO-3 (Molecular Probes) and mounted with fluoromount. Negative controls were prepared by omitting the primary antibody.

Image acquisition was performed with confocal microscope Leica TCS SP2 (Leica, Wetzlar, Germany). Fluorescence signals were quantified by confocal microscope Leica TCS SP2 software and expressed as area fraction (percentage). The number of CD31^+^/α-SMA^+^ and Ki-67^+^/CD31^+^ cells was quantified in at least 10 high-power (×630) fields (HPF)/sections by two independent observers blinded to the origin of the slides. The final counts were the mean of the two measures. In no case was interobserver variability higher than 20%.

### Caspase-3 immunohistochemistry

After epitope unmasking through microwave and citrate buffer, renal tissues were washed with distillated water and incubated with H_2_O_2_ (3%) for 7 minutes at room temperature. They were permeabilized with Triton 0.25% for 5 minutes and blocked with protein block solution (Dako, Glostrup, Denmark) for 10 minutes. Then sections were incubated with the primary antibody (Caspase-3, Novus Biologicals; diluted 1:50) and detected by the Peroxidase/DAB Dako Real EnVision Detection System, according to manufacturer’s instructions (Dako). The peroxidase reaction was shown by a brown precipitate, counterstained with Mayers hematoxylin (blue), and mounted with Glycergel (DakoCytomation, Carpinteria, CA, USA). Negative controls were prepared incubating sections with the blocking solution and then control irrelevant antibody. Digital images were scanned by Aperio ScanScope CS2 device (Aperio Technologies) by two independent observers blinded to the origin of the slides. The selected regions of digital slides were analyzed with the algorithm for analysis of nuclear signal (Immunohistochemistry, IHC, nuclear algorithm, Aperio Technologies) to measure both staining intensity and percentage of positive cells. For each region, the algorithm identified the total number of tubular analyzed cells, and the percentage of cells with absent (0) to strong (3+) nuclear signal. The percentages of positive cells were indicated by the Aperio Software.

### Cell-growth determination kit

Cultured ECs were seeded (2 × 10^4^ cells per well) on 96 wells in 100 μl of complete culture medium.

Cells were treated with LPS, 2 μg/ml, and LPS, 4 μg/ml, for 24 hours. EC proliferation was evaluated by measuring the activity of living cells via mitochondrial dehydrogenase activity by 3-[4,5-dimethylthiazol-2-yl]-2,5-diphenyl tetrazolium bromide or Methylthiazol Tetrazolium (MTT) (Sigma Aldrich).

After 24 hours of stimulation, ECs were incubated with 10% MTT reagent in DMEM for 4 hours. As indicated in manufacturer’s instructions (Sigma Aldrich), mitochondrial dehydrogenases of active and viable EC converted the MTT in formazan crystals. The addition of 100 μl isopropanol per plate dissolved the crystal, generating a purple solution that was measured by spectrophotometer at 570 nm by a microplate reader (DV990BV6,GIO. DE VITA E C. S.R.L, Rome, Italy). The absorbances obtained were compared with an appropriate absorbance/cell number curve. Three independent experiments were performed.

### Detection of viable and apoptotic ECs with flow-cytometry analysis

Apoptotic and viable ECs were evaluated with Annexin V(Ann V)- Fluorescein isothiocyanate (FITC) and propidium iodide (PI) (Beckman Coulter). After 24 hours of LPS 2 μg/ml and 4 μg/ml stimulation, ECs were washed twice with PBS and were removed with trypsin-EDTA. Then 5 × 10^5^ cells were resuspended in 100 μl of ice-cold PBS binding buffer 1× (Beckman Coulter) and incubated with annexin V-FITC and PI for 15 minutes at 4°C in the dark. Finally, 400 μl of PBS binding buffer 1× was added to each tube without washing and analyzed with FC500 (Beckman Coulter) and Kaluza software. This assay was done in triplicate and ECs stimulated for 24 hours with H_2_O_2_, 100 μ*M*, as internal positive control.

### Immunophenotypic analysis

EC phenotype was analyzed with surface staining with the FITC-conjugated anti-CD31 (Miltenyi Biotec), Allophycocyanin (*APC*)- conjugated anti-CD31 (Miltenyi Biotec), phycoerythrin (*PE*)- conjugated anti-VE-cadherin (Biolegend, San Diego, CA, USA), *PE*-conjugated anti-N-cadherin (Biolegend), and intracellular staining with unconjugated anti-vimentin antibody (Abcam) and unconjugated anti-FSP-1 antibody (Abcam).

At the end of the EC stimulation with LPS or swine sera, cells were washed twice with PBS and were removed with PBS-EDTA 2 m*M* and trypsin 0.001×. For surface staining, ECs were resuspended in flow cytometry (FACS) buffer (phosphate-buffered saline, pH 7.2, 0.2% bovine serum albumin, and 0.02% sodium azide) and incubated with FCR blocking reagent (Miltenyi Biotec) for 10 minutes at room temperature.

After blocking incubation, surface markers were added for 15 minutes at 4°C. Then cells were washed with the FACS buffer and were resuspended in each tube with 500 μl of FACS buffer for FACS analysis. Intracellular staining was preceded by fixation and permeabilization with IntraPrep kit (Instrumentation Laboratory) and incubation for unconjugated primary antibody 25 minutes at 4°C. Cells were then washed and labeled with secondary Antibody AlexaFluor 488 (Molecular Probes) for 25 minutes at 4°C. Finally, cells were washed twice and resuspended in FACS buffer for acquisition.

Data were obtained by using a FC500 (Beckman Coulter) flow cytometer and analyzed withKaluza software. Three independent experiments were performed. The area of positivity was determined by using an isotype-matched mAb, and in total, 10^4^ events for each sample were acquired.

### LBP plasma and serum levels

LBP levels were determined in sera and pre/post-cartridge plasma by a commercially available enzyme-linked immunosorbent assay (ELISA; Enzo Life Sciences, Farmingdale, NY, USA) according to manufacturer’s instructions.

### RNA extraction and real-time PCR analysis

Total RNA was isolated with miRNeasy Mini Kit (Qiagen, Hilden, Germany) according to the manufacturer’s instructions, and quantified by NanoDrop ND-1000 Spectrophotometer (NanoDrop Technologies, Inc. Wilmington, DE, USA); its quality was assessed with electrophoresis on the agarose gel (1%). One-half microgram of total RNA was used in a reverse transcription (RT) reaction by using the QuantiTect reverse transcription Kit (Qiagen) according to the manufacturer’s instructions. Quantitative real-time (RT)-PCR was performed on an iCycler Thermal Cycler (Bio-Rad Laboratories, Hercules, CA, USA) by using Collagen I primers (Rev 5′-CAGGGAAGCCTCTCTCTCCT-3′; For 5′-ACGTCCTGGTCAAGTTGGTC-3′, Invitrogen) in combination with SYBR Green dye. The relative amounts of Collagen I mRNA were normalized to GAPDH mRNA as the housekeeping gene.

### Statistical analysis

Data were expressed as median ± interquartile range (IQR) and compared with a Mann–Whitney test. For FACS analysis, MTT assay and real-time PCR, data were shown as mean ± standard deviation (SD) or standard error of the mean (SEM) and compared with the Student *t* test or ANOVA, as appropriate. A *P* value <0.05 was considered statistically significant. All analyses were performed by using GraphPad Prism 5.0 (GraphPad software, Inc., San Diego, CA, USA).

## Results

### Endotoxemia-induced oliguric kidney injury is associated with early development of tissue fibrosis

**Figure 1:**
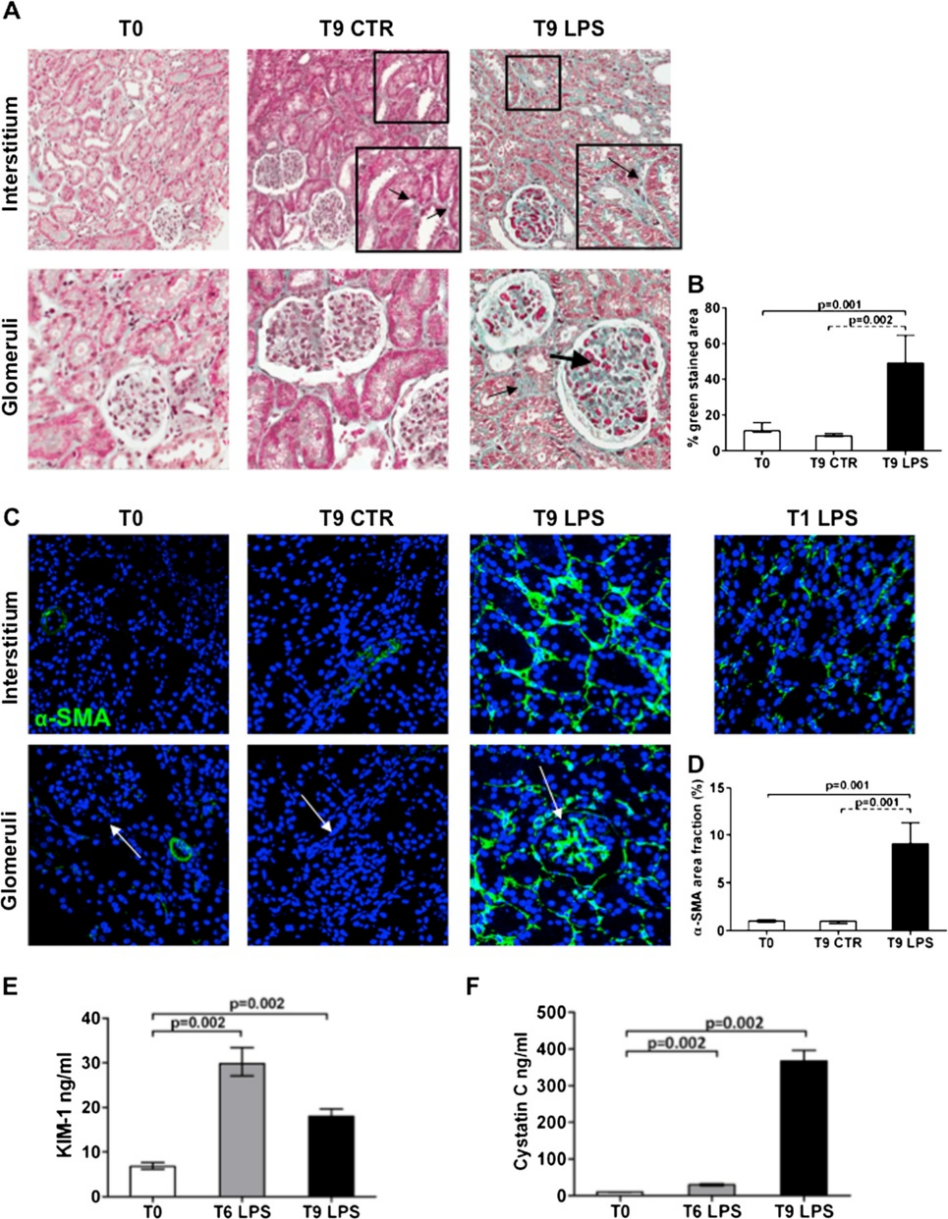
**LPS-induced AKI is associated with early development of tissue fibrosis.** In a swine model of LPS-induced AKI, Masson trichrome staining **(A)** revealed extensive collagen deposition at the interstitial level (thin black arrow), along capillaries (zoomed image), and diffuse glomerular thrombi (thick black arrow) after 9 hours from LPS infusion compared with T0 and T9 of the control group. The pictures are displayed at 4× magnification (**A**, first line) and 10× magnification (**A**, second line). **(C)** In basal condition, no α-SMA^+^ cells (green) were localized in the interstitium and within glomeruli (white arrows). A dramatic increase in α-SMA expression was observed at tubulointerstitial level and within glomeruli and the Bowman capsule (white arrow). An early tubulointerstitial expression of α-SMA was found already at 1 hour with LPS injection. Magnification 630×. To-pro 3 was used to counterstain nuclei (blue). The quantitative analyses of Masson trichrome **(B)** and α-SMA staining **(D)** were obtained as described in the Methods section and expressed as median ± IQR of at least five independent pigs for each group. The ELISA tests **(E, F)** showed a significant increase of KIM-1 and cystatin C in swine sera at 6 and 9 hours from LPS administration compared with T0.

To characterize the possible development of renal fibrosis, we first analyzed kidney biopsies with Masson trichrome staining (Figure 1A). Control animals did not present significant tubulointerstitial and glomerular collagen deposits both at T0 and 9 hours after the infusion of control saline solution. On the contrary, LPS infusion induced an extensive collagen deposition at tubulointerstitial level, in particular, surrounding peritubular capillaries. Endotoxemic pigs showed diffuse glomerular thrombi and tubular vacuolization that were not detectable in control animals. Quantification of stainings indicated that LPS-treated animals had a statistically significant increase in collagen deposits compared with controls (Figure 1B).

Next we investigated the expression of α-SMA, a marker of activated myofibroblasts. We detected a significant expansion of α-SMA^+^ cells in endotoxemic pigs, particularly at tubulointerstitial level (Figure 1C) and within glomeruli. As expected, in normal conditions, this marker was almost absent both at tubulointerstitial and glomerular level; α-SMA was associated only with smooth muscle cells of renal arteries. Interestingly, tubulointerstitial α-SMA expression was present already 1 hour after LPS infusion. The overall increase in α-SMA expression (D) in endotoxemic animals was statistically significant when compared with controls.

Finally, we investigated whether the development of acute fibrosis was associated with the expression of biomarkers of tubular damage (Figure 1E, F). After 6 and 9 hours from LPS injection, endotoxemic animals showed a significant increase in serum levels of KIM-1 and cystatin C, compared with the basal condition. On the contrary, serum creatinine was similar in endotoxemic pigs and controls (data not shown).

**Figure 2:**
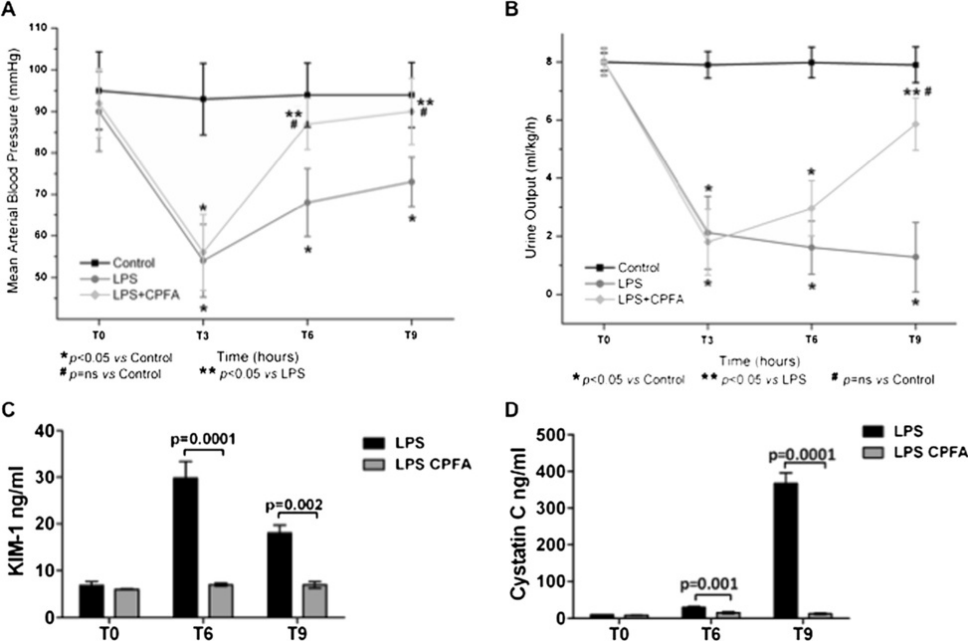
**Recovery of hypotension, oligoanuria, and renal function in endotoxemic animals. (A)** Endotoxemic pigs developed hypotension after 3 hours of LPS infusion. CPFA treatment restored blood pressure to basal condition. **(B)** Presence of oligoanuria was a clear sign of LPS-induced AKI. Urinary output was significantly reversed after 6 hours of CPFA treatment. CPFA treatment significantly reduced serum KIM-1 **(C)** and cystatin C **(D)** at 6 and 9 hours from LPS infusion.

In addition, LPS-treated pigs developed hypotension (Figure 2A). Considering the normal urinary output in pigs (5 to 30 ml/kg/h), the urinary output in our model during endotoxemia reached a value of 1.5 ml/kg/h, indicating the occurrence of oliguric kidney injury (Figure 2B).

### Association of endothelial dysfunction with acute development of renal fibrosis

**Figure 3:**
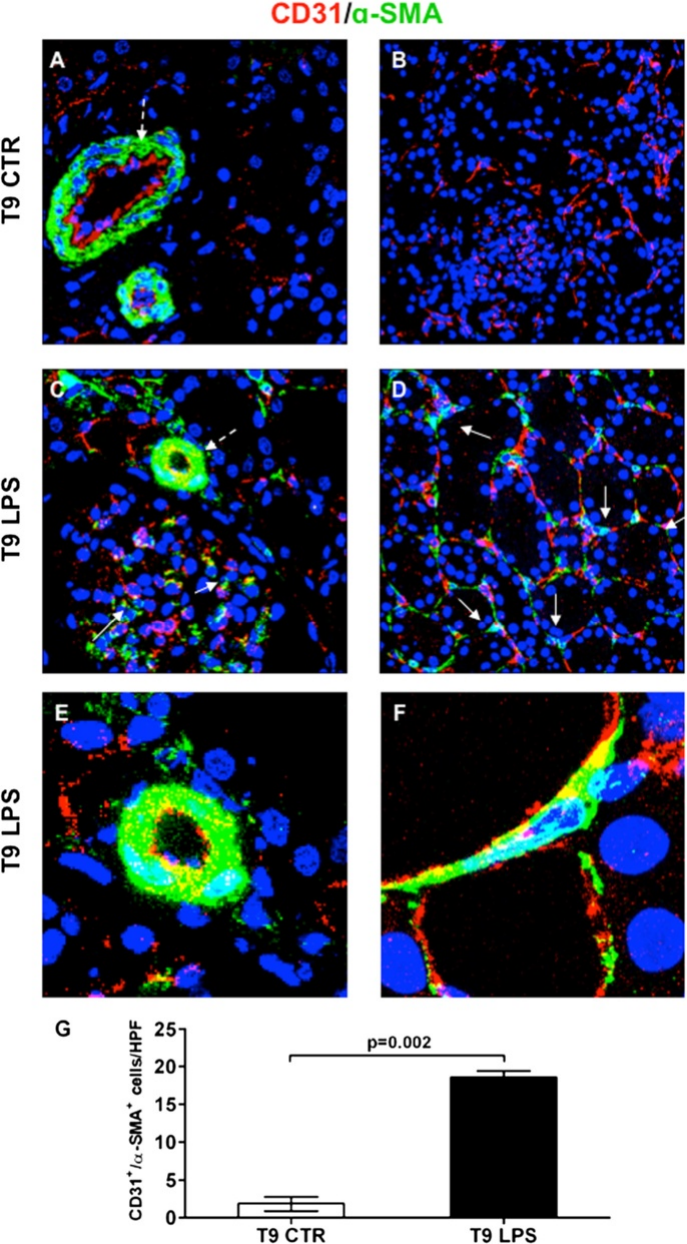
**Characterization of renal EC dysfunction** ***in vivo*** **.** ECs were double-stained for CD31 (red) and α-SMA marker (green) further to demonstrate the occurrence of endothelial dysfunction. In renal vessels of endotoxemic tissue (**C**, dotted white arrow), CD31^+^EC expressed α-SMA marker and migrated from the intima to the media of the vessel wall (**E**, zoomed image), in otherwise basal condition (**A**, dotted white arrow). In the interstitium of T9 control pig **(A, B)** CD31^+^/α-SMA^+^ cells were rarely detectable. After 9 hours from LPS infusion, the number of these cells increased dramatically within glomeruli (**C**, white arrows) and interstitium (**D**, white arrows). Zoomed image of EC **(F)** co-expressing CD31 and α-SMA in endotoxemic tissue. Results are expressed as median ± IQR of the numbers of CD31^+^/α-SMA^+^ cells/HPF of at least five independent pigs for each group **(G)**. Magnification, 630×. To-pro 3 was used to counterstain nuclei (blue).

We then performed a double immunofluorescence staining for EC marker CD31 and α-SMA. In control condition, EC showed a clear distribution of CD31 both in the intima of the renal arteries (Figure 3A) and in peritubular capillaries (Figure 3B). After LPS infusion, the phenotype of renal EC dramatically changed. In renal vessels (Figure 3C, E), CD31^+^ EC acquired α-SMA and could be detected in the media of the vascular wall, indicating a possible migration from intima to media (Figure 3E). Moreover, we found a significant increase in peritubular α-SMA^+^ EC (Figure 3D). The colocalization of these two markers on ECs was statistically significant in LPS-treated animals (Figure 3G) and occurred particularly at the tubulointerstitial level (Figure 3F).

### Phenotypic characterization of dysfunctional ECs in endotoxemia

**Figure 4:**
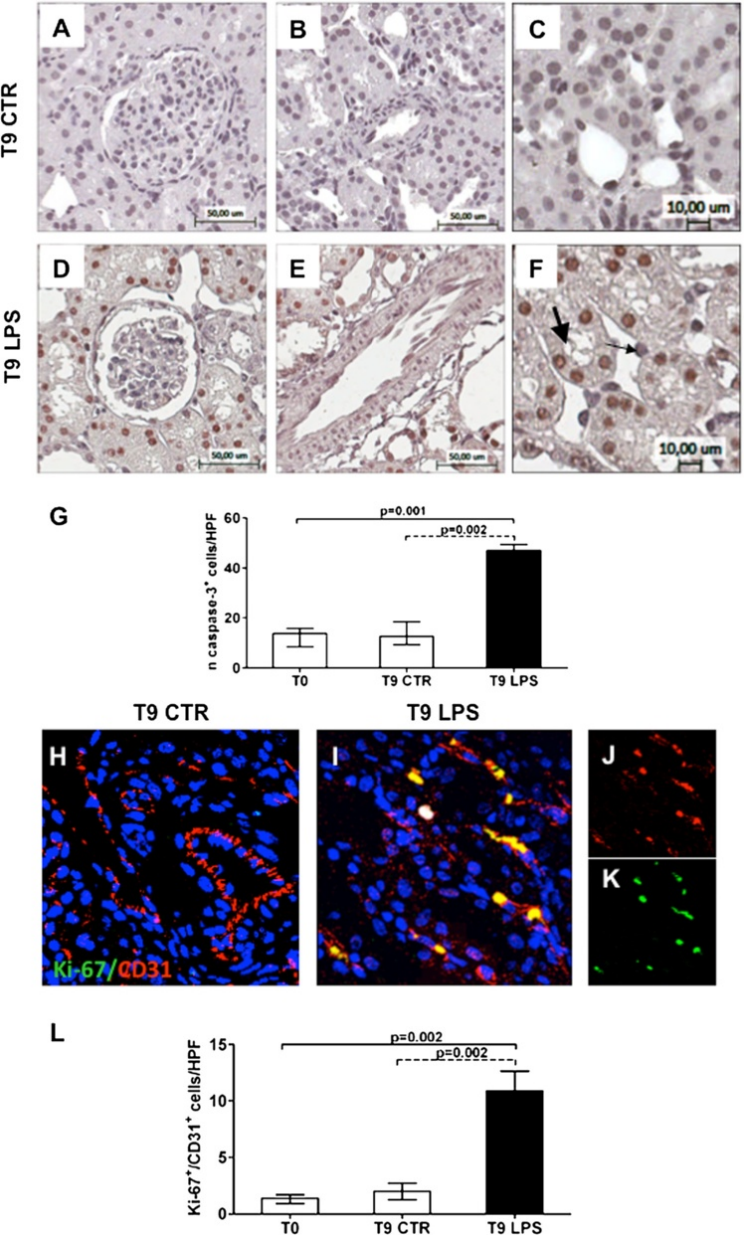
**Characterization of dysfunctional ECs in endotoxemia.** Immunohistochemical analysis for caspase-3 showed rare apoptotic EC in endotoxemic pigs within glomeruli **(D)**, at vascular **(E)** and peritubular levels (**F**, zoomed image, thin black arrow). Moreover, an increased number of tubular cells underwent apoptosis (F, zoomed image, thick black arrow). Caspase-3 nuclear expression in tubular cells **(G)** was analyzed as reported in the Methods section and expressed as median ± IQR of at least five independent pigs for each group. Magnification 10× **(A, B, D, E)** and 20× **(C, F)**. Double immunofluorescence analysis (CD31 red; Ki-67 green) revealed Ki-67^+^/CD31^+^ proliferating ECs in endotoxemic pigs **(I)** compared with the T9 control group **(H)**. Single-color images **(J, K)** underlined the co-localization of C31 (red J) and Ki-67 (green K) markers on ECs. Results are expressed as median ± IQR of the numbers of Ki-67^+^/CD31^+^ cells/HPF of at least five independent pigs for each group **(L)**. Magnification, 630×. To-pro 3 was used to counterstain nuclei (blue).

Next we wanted to characterize the occurrence of apoptosis in dysfunctional ECs by caspase-3 staining. Caspase-3 expression was barely detectable in vehicle-treated animals (Figure 4A-C), whereas renal tubular epithelial cells of endotoxemic pigs were strongly positive (Figure 4F). The difference between the two experimental conditions was statistically significant (Figure 4G). Conversely, ECs at vascular and glomerular levels were not involved in apoptotic processes (Figure 4D, E). In addition, we studied whether CD31^+^ ECs expressed a marker of cellular proliferation. Compared with normal conditions (Figure 4H), we found a significant increase of Ki-67^+^/CD31^+^ ECs in endotoxemic tissues (Figure 4I-K). Proliferating ECs in endotoxemic pigs were significantly higher compared with controls (Figure 4L).

### LPS induced EC dysfunction *in vitro* with collagen I mRNA synthesis

**Figure 5:**
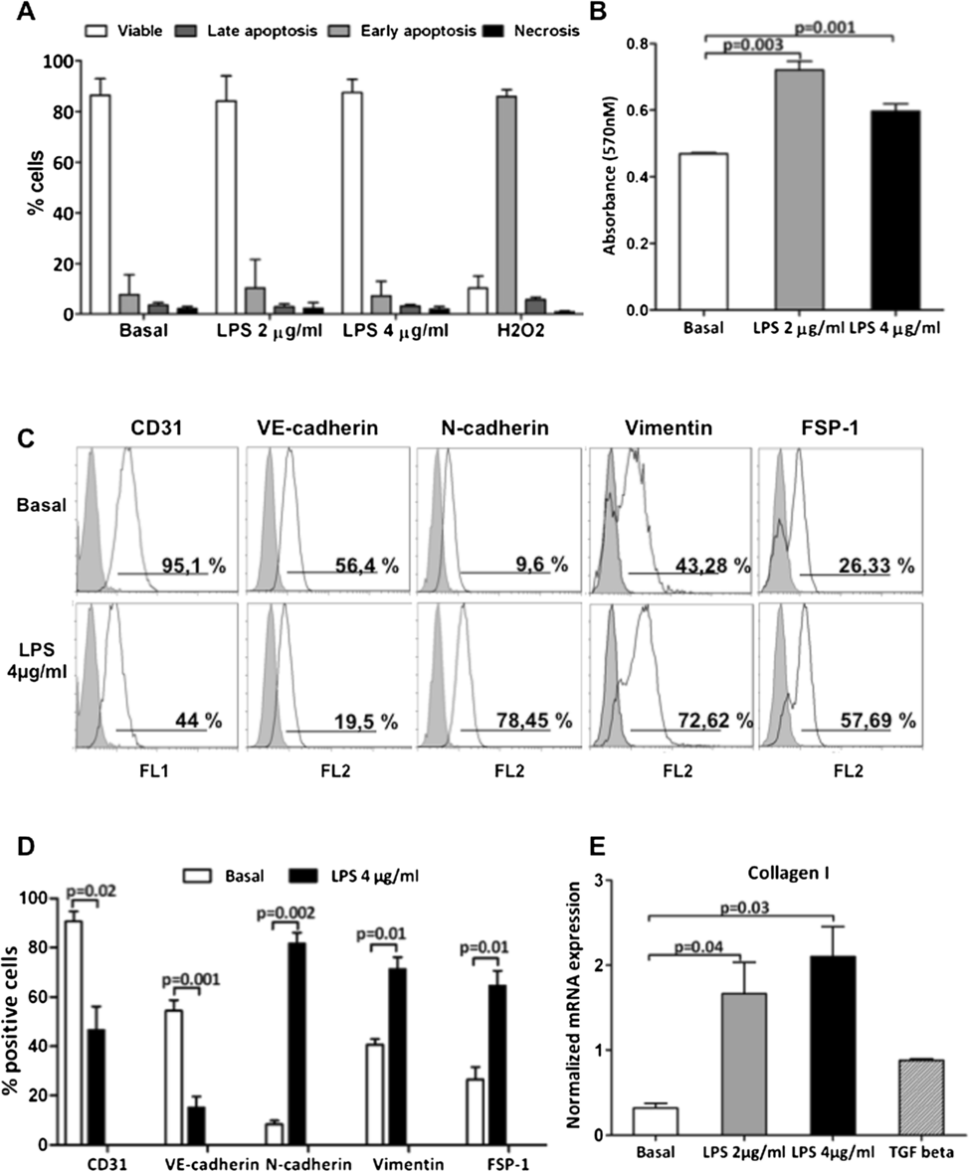
**LPS induced EC dysfunction and collagen I mRNA synthesis.** Cultured ECs were incubated with LPS, 2 μg/ml, or LPS, 4 μg/ml, for 24 hours. **(A)** EC viability was evaluated with FACS analysis (AnnV/PI). Only a small percentage of ECs underwent apoptosis after LPS, 2 μg/ml (8.9% ±1.6 versus basal 5. 9% ±1.2) and LPS, 4 μg/ml (7.8% ±1.6) stimulation compared with ECs treated with H_2_O_2_ 100 μ*M* for 24 hours (positive control). **(B)** MTT cell-viability assay highlighted a significant proliferation of ECs after LPS, 2 μg/ml (0.720 ± 0.03 versus basal 24-hour 0.469 ± 0.004; *P* = 0.03) and LPS 4 μg/ml (0.597 ± 0.03 versus basal 24 hour 0.469 ± 0.004, *P* = 0.04) stimulation. **(C, D)** FACS analysis of EC showed the phenotypic changes induced by 24 hours of LPS stimulations. Results are expressed as mean ± SD and are representative of three independent experiments. **(E)** Real-time RT-PCR revealed the mRNA expression levels of collagen I. The gene relative expression was normalized to the expression of GAPDH. The histograms represent the mean ± SEM and are representative of three independent experiments.

We then investigated whether LPS could directly induce EC dysfunction *in vitro*. After 24 hours of LPS activation, FACS analysis showed that only a small percentage of ECs underwent apoptosis (Figure 5A). By MTT cell viability assay, we observed a significant proliferation of EC when activated by LPS (Figure 5B). The FACS analysis showed that control ECs presented, as expected, high levels of EC markers, CD31 and VE-cadherin, and low levels of EC dysfunction markers, such as N-cadherin, vimentin, and FSP-1.

After 24 hours of incubation, LPS induced a significant reduction in CD31 and VE-cadherin expression, with a concomitant dose-dependent increase of N-cadherin, vimentin and FSP-1 (Figure 5C, D). Moreover, after 6 and 9 hours from LPS exposure, α-SMA^+^ ECs were barely detectable (data not shown).

Finally, LPS incubation induced a significant increase in collagen I mRNA synthesis, indicating that EC dysfunction resulted in active contribution to the synthesis of extracellular matrix components (Figure 5E).

### Prevention of LPS-induced renal fibrosis by citrate-based CPFA treatment

Next we tested whether CPFA might counteract the detrimental effects of LPS and the development of renal fibrosis *in vivo*.

**Figure 6:**
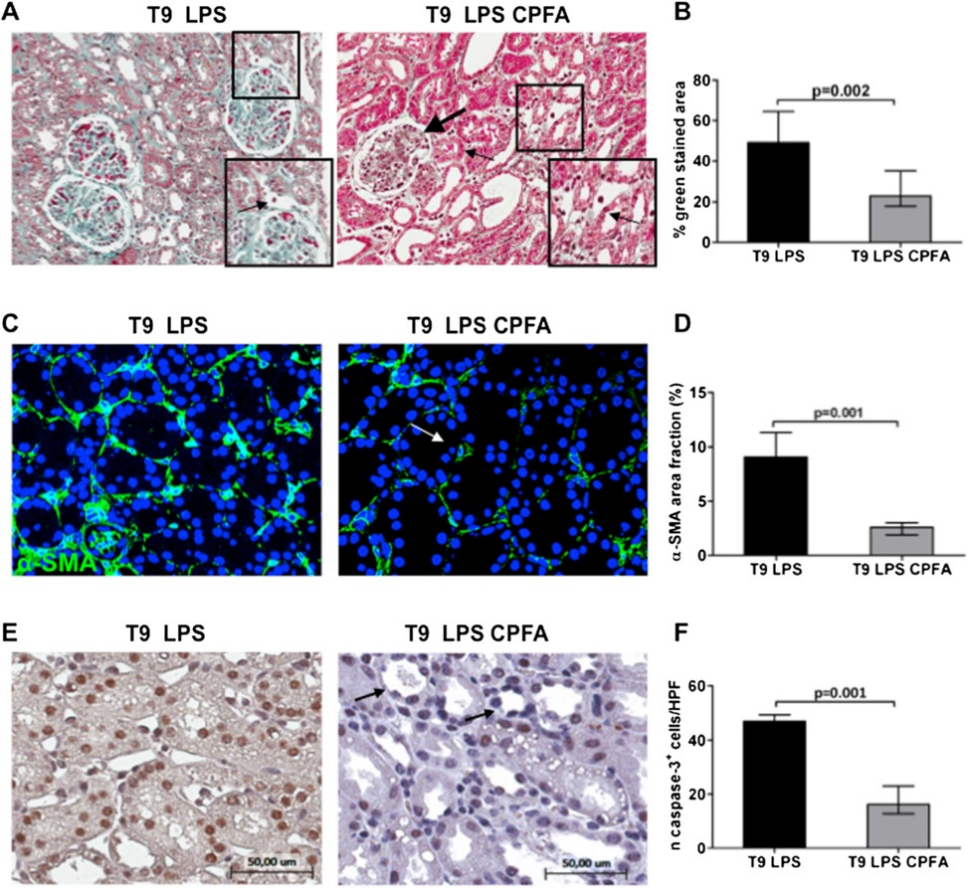
**Prevention of LPS-induced renal fibrosis by CPFA treatment.** Renal biopsies after CPFA treatment showed a significant reduction in collagen deposits (**A**, thin arrow), in particular along capillaries (zoomed image), and glomerular thrombi (thick arrow) compared with endotoxemic condition. Magnification 6×. **(C)** Immunofluorescence analysis showed a strong expression of interstitial α-SMA (green) in endotoxemic pigs that was dramatically decreased by CPFA treatment (white arrow). Magnification, 630×. To-pro 3 was used to counterstain nuclei (blue). **(E)** Caspase-3 staining highlighted the efficacy of treatment (black arrows) in reversing LPS-induced tubular apoptosis. Magnification, 14×. The quantitative analyses of Masson trichrome **(B)**, α-SMA **(D),** and caspase-3 **(F)** staining were obtained as described in the Methods section and expressed as median ± IQR of at least five independent pigs for each group.

After 3 hours from LPS infusion, endotoxemic animals were treated for 6 hours with CPFA; renal biopsies were performed at T9. Masson trichrome staining showed that treatment significantly reduced renal fibrosis, as shown by decreased collagen deposits compared with endotoxemic pigs (Figure 6A, B). The reduction of extracellular matrix deposits was particularly evident at peritubular capillary level. We observed very limited glomerular thrombi compared with untreated animals. The induction of peritubular α-SMA expression (Figure 6C, D) and tubular apoptosis (Figure 6E, F) were also significantly hampered in treated pigs. Moreover, arterial blood pressure and urinary output significantly improved after 6 hours of treatment (Figure 2A, B). Finally, the downregulation of serum biomarkers of renal injury, KIM-1 and cystatin C, demonstrated a protective effect of CPFA on tubular damage (Figure 2C, D).

### Inhibition of EC dysfunction by CPFA treatment

**Figure 7:**
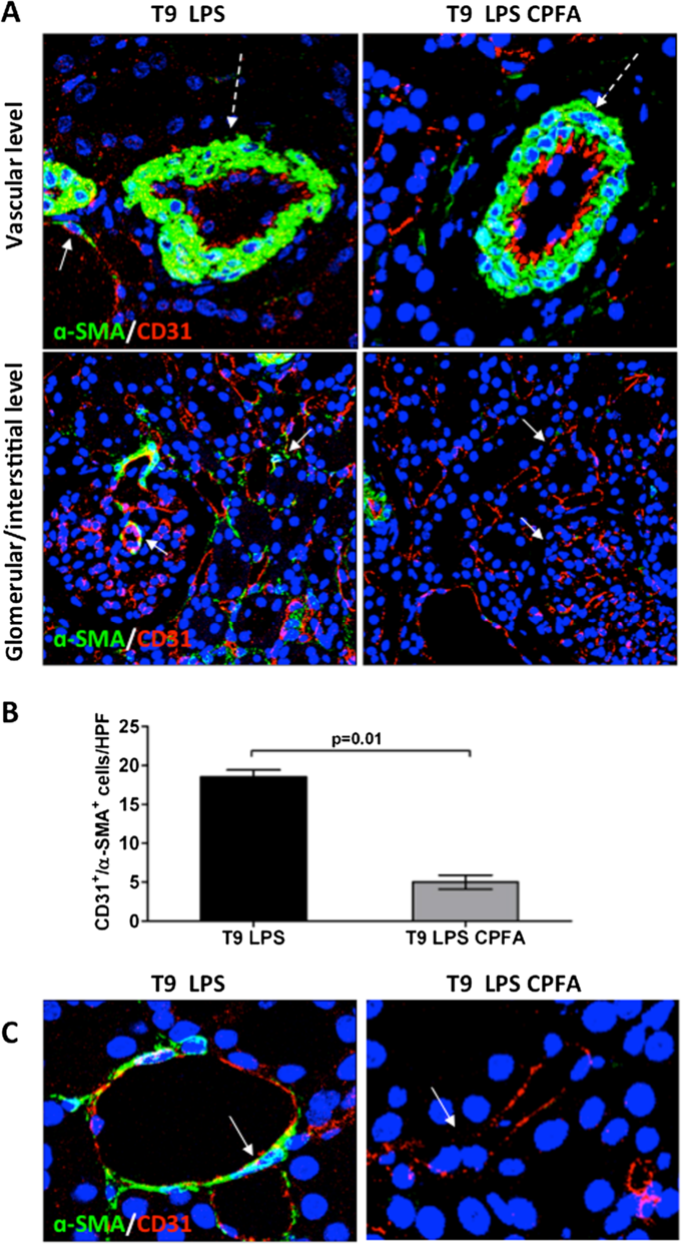
**Inhibition of EC dysfunction by CPFA treatment. (A)** In endotoxemic animals, the reduced CD31 (red) expression at vascular level (dotted white arrow) was reversed after 6 hours of CPFA treatment (dotted white arrow). The increased number of CD31^+^/α-SMA^+^ cells induced by LPS infusion (white arrows) was significantly hampered in treated pigs (white arrows). **(B)** Results are expressed as median ± IQR of the numbers of CD31^+^/α-SMA^+^ cells/HPF of at least five independent animals for each group. **(C)** Comparison of zoomed peritubular capillaries of endotoxemic (white arrow) and treated endotoxemic pigs (white arrow). Magnification, 630×. To-pro 3 was used to counterstain nuclei (blue).

Then we investigated the effects of CPFA on EC dysfunction. As indicated in Figure 7A, renal arteries showed a preservation of CD31 expression on vessel intima and the absence of CD31^+^/α-SMA^+^ cells in the vessel media compared with endotoxemic pigs. In accordance, the number of dysfunctional CD31^+^/α-SMA^+^ cells in the tubulointerstitium of endotoxemic pigs was significantly reduced by CPFA treatment (Figure 7B).

### Removal of LPS-binding protein from plasma by CPFA treatment

**Figure 8:**
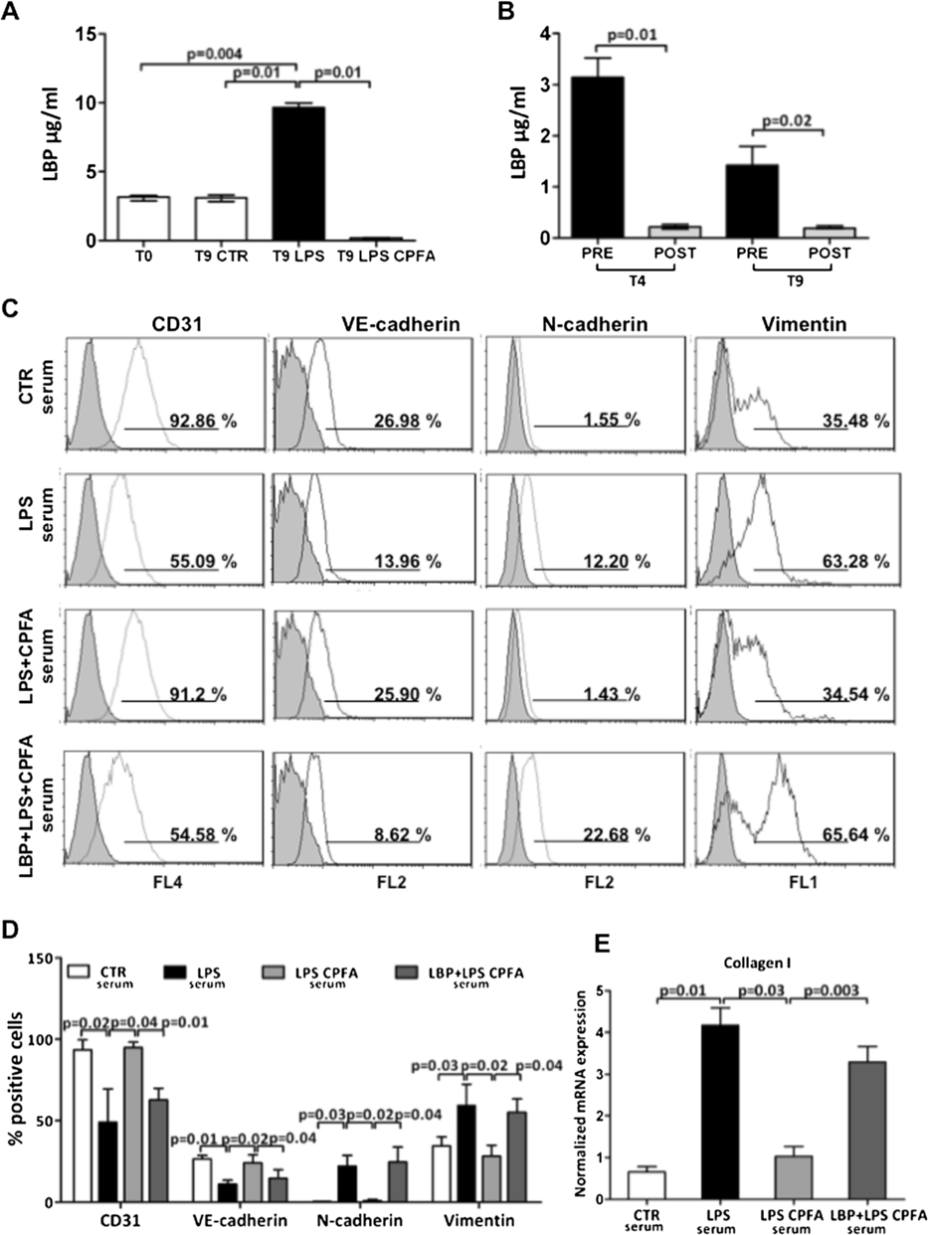
**LBP is pivotal in LPS-mediated endothelial dysfunction. (A)** ELISA revealed the increased level of LBP in sera of endotoxemic pigs comparing with the control group at T0 and T9. A considerable reduction in serum LBP levels was found after 6 hours of CPFA treatment. **(B)** Plasma samples drawn from the CPFA circuit were also analyzed. A significant decrease of LBP was found in the plasma filtrate effluent from the sorbent cartridge 1 hour after circuit installation (T4 plasma postcartridge). After 6 hours from the start of treatment, LBP levels in plasma postcartridge (T9 plasma postcartridge) were lower than in plasma precartridge. The histograms represent the median ± IQR of at least five independent animals for each group. **(C-E)** Cultured ECs were treated with sera of healthy (CTR), endotoxemic (LPS), and CPFA-treated endotoxemic (LPS CPFA) pigs. **(C, D)** FACS analysis of ECs showed phenotypic changes after 12 hours of LPS sera incubation. In the presence of LPS CPFA sera, EC preserved their phenotypes. After LBP addition in LPS CPFA sera, ECs showed phenotypic changes (LBP + LPS CPFA). **(D)** Results are representative of three independent experiments. **(E)** The expression of collagen I was detected by real-time RT-PCR. The gene relative expression was normalized to the expression of GAPDH. The histograms represent the mean ± SEM and are representative of three independent experiments.

We then investigated whether the protective effect of CPFA treatment was due to possible modulation of LBP, a soluble compound of the LPS/TLR-4 complex. The serum levels of LBP, an acute-phase protein, significantly increased after 9 hours of LPS infusion compared with controls (Figure 8A). Interestingly, CPFA-treated pigs presented a dramatic reduction in serum LBP levels. When we measured the concentration of LBP before and after the plasma passage throughout the sorbent cartridge at different time points, we observed a significant decrease in LBP concentration in the plasma effluent from the sorbent cartridge already 1 hour after starting treatment (Figure 8B, T4). After 6 hours of treatment, LBP concentration further decreased before and after the sorbent cartridge, reaching very low levels in the postcartridge sample (Figure 8B, T9). Despite the removal of serum LBP, endotoxin levels remained unchanged (LAL Test, data not shown).

### LBP is pivotal in LPS-mediated endothelial dysfunction in sepsis

Finally, we investigated whether the removal of LBP by CPFA was critical in mediating EC dysfunction in LPS-treated animals. We cultured ECs in the presence of different pig sera for 12 hours. FACS analysis (Figure 8C, D) showed that serum from endotoxemic pigs treated with CPFA prevented EC dysfunction, with higher CD31 and VE-cadherin expression compared with endotoxemic pigs. In accordance, CPFA treatment maintained the expression of the dysfunctional markers N-cadherin and vimentin at low levels. Remarkably, the addition of exogenous LBP in sera from CPFA-treated endotoxemic pigs induced EC dysfunction like that of the untreated endotoxemic animals sera.

When we quantified the relative expression of all the markers tested, we found that the LBP supplementation completely reversed the effects of CPFA-treated endotoxemic pig serum, leading to EC dysfunction (Figure 8D). Exogenous LBP was also capable of restoring the capacity of CPFA-treated endotoxemic sera to induce collagen I mRNA synthesis in ECs (Figure 8E).

## Discussion

In this study, we demonstrated that endotoxemia-induced oliguric kidney injury is characterized by an acute development of renal fibrosis, associated with endothelial dysfunction. In our model, CPFA treatment could significantly limit renal fibrosis and EC dysfunction by an efficient removal of the LPS-adaptor protein LBP.

Sepsis has been well recognized as a systemic inflammatory response to an active infectious process in the host [16], which frequently leads to multiorgan dysfunction, shock, and death [3]. Among the several disorders encountered in sepsis, the incidence of AKI remains very high, and an appropriate therapeutic strategy has not been identified [17]. The pathophysiology of AKI in sepsis is very different from nonseptic AKI [18] and is characterized by alterations in the homeostasis of immune, coagulation, and cardiovascular systems. Sepsis-induced AKI can be driven by different mechanisms [9]. In particular, the LPS/TLR-4 axis plays a pivotal role in this setting [19]. When the TLR-4 pathway was blocked *in vivo*, a significant protection occurred in animal models of LPS-induced AKI [20]. Accordingly, humans lacking myeloid differentiation protein 88 (MyD88) activity had very limited severe infections [21]. Interestingly, LPS could modify systemic and local cytokine production with significant alteration in peritubular capillary networks [19]. During sepsis, ECs may switch from a quiescent to an activated state, more commonly defined as endothelial dysfunction [6]. In this phase, ECs may undergo structural changes, acquiring dysfunctional markers with a fibroblast-like phenotype [22], with possible development of fibrosis [23,24]. Dysfunctional ECs promoted leukocyte adhesion and trafficking along with altered vascular tone [25], disseminated intravascular coagulation, and glomerular microthrombi [19,26].

After LPS binding on ECs, the TLR-4 signaling involves the activation of the NF-kB, mitogen-activated protein kinase (MAPK) and phosphatidylinositol 3-kinase (PI3K)/Akt pathways that regulate the balance between cell viability and inflammation. In particular, PI3K/Akt pathway was identified as the principal survival signal in LPS-treated ECs [27]. A recent study demonstrated that LPS protected microvascular lung ECs from apoptosis [12]. Our data confirmed that LPS-induced EC dysfunction is not associated with apoptotic ECs both *in vivo* and *in vitro*. On the contrary, we found a significant apoptosis of tubular epithelial cells that occurred early, as in other animal models [21]. In addition, our model is in line with the histologic data obtained in sepsis patients with no evidence of acute tubular necrosis but described an increased vacuolization and flattening of the brush border [21,28].

Next to tubular damage and endothelial dysfunction, we observed the acute development of tubulointerstitial fibrosis. To our knowledge, the early development of renal fibrosis in our model might be a feature of swine, because it could be detected also in ischemia/reperfusion-induced AKI [29], and it has never been found in rodent models [21]. Inflammation usually precedes fibrosis, that probably initiates as a beneficial mechanism of repair [6]. Conversely, a persistent injury results in a pathologic condition, typical of all progressive renal diseases, with massive deposition of extracellular matrix [30], tubular atrophy and dilatation, tubulointerstitial fibrosis, glomerulosclerosis, and endothelium damage.

Our histologic data are in line with the recent evidence regarding the role of bacteria and TLR in the pathogenesis of fibrosis [30]. TLR-4 was recently recognized as a significant mediator of fibroblast accumulation and tubulointerstitial fibrosis during renal injury [31,32]. Microbial products might also regulate the production of TGF-β and oxidative stress [30], with a significant impact on fibrogenesis.

Interestingly, renal EC might lead to development of renal fibrosis by direct and indirect mechanisms [6]. Peritubular capillaries constitute the major network supplying oxygen to the nephrons. Therefore, alterations in renal ECs might induce progressive hypoxia of renal tubular epithelial cells with alteration in prostaglandin synthesis and generation of reactive oxygen species [7]. Morphologic alterations of peritubular capillaries were strongly associated with parameters of tubulointerstitial injury in humans [33]. Moreover, recent evidence supports a direct effect of ECs in fibrogenesis. A particular form of EC dysfunction is the endothelial-to-mesenchymal transition that may lead to the generation of a certain percentage of renal fibroblasts [24] in chronic kidney disease [23]. In our model, dysfunctional ECs acquired several myofibroblasts markers and produced collagen I when activated by LPS *in vitro* and *in v*i*vo*, therefore acquiring multiple functions of myofibroblasts. In addition, the development of EC dysfunction in our model *in vivo* required only 9 hours, whereas 24 hours was necessary by LPS stimulation on ECs *in vitro*. Interestingly, we found transitioning CD31^+^/α-SMA^+^ ECs in the media of renal vessels, which might have migrated from the intima. These data are in line with other works describing the invasiveness capacity of ECs after transition to the mesenchymal state, a process named endothelial-to-mesenchymal transition [24]. The reduction in CD31 staining was limited to ECs localizing only in renal vessels, indicating the presence of different factors influencing the activation of ECs. In this context, smooth muscle vascular cells might be pivotal in driving the development of these transitioning ECs, by producing interleukins, chemokines, and growth factors [34].

Modulation of the host response to endotoxin is one of the major therapeutic targets in sepsis-induced AKI [35]. However, the use of immunotherapy to modify potential sites of the LPS/TLR-4 signaling pathway showed insufficient effects [36]. Extracorporeal blood-purification therapies have been proposed to improve outcomes of sepsis patients [18]. Our model of LPS-induced AKI, despite the lack of a precise dissection of the molecular pathways described in mice, is characterized by a supportive therapy comparable to the standard for ICU patients, which enables us to evaluate additional benefits beyond the conventional fluid-infusion therapy [21]. However, a limitation in the present study is represented by the lack of a blinded collection of tissues and biologic samples in the different experimental conditions.

CPFA is a modality of blood purification in which plasma circulates in a sorbent cartridge. In CPFA treatment, plasma is separated from the whole blood and, after passing through the sorbent, it is reinfused into the blood circuit reconstituting the whole blood structure without proinflammatory but also antiinflammatory cytokines [17,37]. The use of citrate provides anticoagulation of the extracorporeal-circuit blood by chelating ionized calcium [38], with potential protective effects against endothelial inflammation and dysfunction [39].

In our model, we observed a beneficial effect of CPFA treatment. However, it is important to consider that because CPFA cannot remove LPS from the circulation [37] because of the features of the sorbet cartridge, we hypothesized that the beneficial effects in our model might be due to the clearance of another key mediator of LPS signaling. LPS-induced cell activation *in vivo* depends on the presence of at least four proteins: TLR-4, MD-2, CD14, and LBP [10,11,40–43]. LBP facilitates the uptake of LPS and LPS-CD14 complexes into ECs and tubular cells [11]. Considering the molecular mass of 60 kDa that was in the range of the adsorptive capacity of the CPFA cartridge, we focused our attention on LBP, a soluble carrier of LPS. LBP can trigger an inflammatory response to chronic or recurring low LPS [10]. The deleterious effects of LPS can occur at low concentrations in the presence of LBP that can also facilities the uptake of endotoxins within the cells [10,44–47].

In our study, CPFA treatment was critical to maintain LBP at a low level with significant protective effect on EC dysfunction, both *in vivo* and *in vitro*. These data support the hypothesis that the possible elimination of cytokine-modulating factors rather than cytokines themselves should also be considered when dealing with these types of treatments. Therefore, considering our data, it is possible to postulate a scenario in which a selective removal of LBP might be sufficient to block the detrimental effect of LPS *in vivo*, including LPS-mediated systemic and local cytokines release.

## Conclusions

In conclusion, we demonstrated that in the early phase of LPS-induced AKI, renal fibrosis is accompanied by α-SMA^+^ dysfunctional ECs. Extracorporeal treatment by CPFA protects renal parenchyma by preventing the development of tubulointerstitial fibrosis, tubular apoptosis, and EC dysfunction. Finally, we identified, in the removal of LBP, the critical factor that can eliminate the effect of LPS in our model. Considering that renal fibrosis is pivotal in the development of renal failure, we hypothesize that a selective removal of LBP might represent a future therapeutic strategy with a significant impact on short- and long-term outcomes for patients with LPS-induced AKI.

## Key messages

- Endothelial dysfunction and early renal fibrosis might have a central role in the pathogenesis of endotoxemia-induced oliguric AKI.
- When activated by LPS, ECs proliferated and acquired several markers of myofibroblasts, therefore contributing to the development of fibrosis and tissue injury.
- CPFA reduced renal fibrosis, tubular apoptosis, and endothelial dysfunction, preserving renal tissue damage.
- Selective removal of LBP might be sufficient to block the detrimental effect of LPS *in vivo*, and might represent a future therapeutic strategy.

## Abbreviations

AKI: Acute kidney injury
CPFA: coupled plasma filtration adsorption
EC: endothelial cell
HPF: high-power field
IQR: interquartile range
KIM-1: kidney injury molecule-1
LBP: LPS-binding protein
LPS: lipopolysaccharide
MTT: methylthiazol tetrazolium
SD: standard deviation
SEM: standard error of the mean
TLR-4: Toll-like receptor-4
